# Comparison between First- and Second-Generation Cryoballoon for Paroxysmal Atrial Fibrillation Ablation

**DOI:** 10.1155/2016/5106127

**Published:** 2016-03-16

**Authors:** Sergio Conti, Massimo Moltrasio, Gaetano Fassini, Fabrizio Tundo, Stefania Riva, Antonio Dello Russo, Michela Casella, Benedetta Majocchi, Vittoria Marino, Pasquale De Iuliis, Valentina Catto, Salvatore Pala, Claudio Tondo

**Affiliations:** ^1^Cardiac Arrhythmia Research Centre, Centro Cardiologico Monzino IRCCS, Via Carlo Parea 4, 20138 Milan, Italy; ^2^St. Jude Medical, Agrate Brianza, Italy

## Abstract

*Introduction*. Cryoballoon (CB) ablation has emerged as a novel treatment for pulmonary vein isolation (PVI) for patients with paroxysmal atrial fibrillation (PAF). The second-generation Arctic Front Advance (ADV) was redesigned with technical modifications aiming at procedural and outcome improvements. We aimed to compare the efficacy of the two different technologies over a long-term follow-up.* Methods*. A total of 120 patients with PAF were enrolled. Sixty patients underwent PVI using the first-generation CB and 60 patients with the ADV catheter. All patients were evaluated over a follow-up period of 2 years.* Results*. There were no significant differences between the two groups of patients. Procedures performed with the first-generation CB showed longer fluoroscopy time (36.3 ± 16.8 versus 14.2 ± 13.5 min, resp.; *p* = 0.00016) and longer procedure times as well (153.1 ± 32 versus 102 ± 24.8 min, resp.; *p* = 0.019). The overall long-term success was significantly different between the two groups (68.3 versus 86.7%, resp.; *p* = 0.017). No differences were found in the lesion areas of left and right PV between the two groups (resp., *p* = 0.61 and 0.57). There were no significant differences in procedural-related complications.* Conclusion*. The ADV catheter compared to the first-generation balloon allows obtaining a significantly higher success rate after a single PVI procedure during the long-term follow-up. Fluoroscopy and procedural times were significantly shortened using the ADV catheter.

## 1. Introduction

Pulmonary vein isolation (PVI) is the cornerstone of any catheter-based treatment for patients with paroxysmal atrial fibrillation (PAF) [1, 2]. Electrical isolation is commonly performed by a circumferential lesion set around the pulmonary veins [1–3]. The standard “point-by-point” technique remains challenging and time-consuming. Cryoballoon (CB) technology would theoretically allow PVI with a single application [4–8]. The first-generation CB, Arctic Front*™* (Medtronic, Inc., Minneapolis, MN, USA), has been available since 2006 in Europe [7, 8]. With respect to the first-generation CB, the second-generation, Arctic Front Advance*™* (ADV), version was designed with technical modifications aiming at procedural outcome improvement [9–11]. The number of injection ports has been doubled and these have been placed more distally on the catheters shaft resulting in a larger and more uniform zone of freezing on the balloons surface if compared with the previous version [12]. Aim of the study was to compare the acute and long-term success of these two different technologies.

## 2. Methods

### 2.1. Patient Population

We retrospectively analyzed 120 patients undergoing PVI using the CB technology who completed at least 2 years of follow-up. All patients had symptomatic and drug-resistant PAF according to the current ESC and HRS/EHRA/ECAS guidelines [1, 2]. Data were accurately collected for each patient from medical notes after discharge and included basic demographic, clinical information, pharmacological therapy, date of hospitalization and discharge, presence of comorbidities, and cardiovascular events during hospitalization. From June 2011 to June 2013 sixty patients underwent PVI using the Arctic Front*™* CB catheter and 60 patients using the ADV ablation catheter. The 28 mm CB was used in all procedures. In addition, electroanatomical mapping using NavX Velocity 3.0 system (St. Jude Medical, Minneapolis, MN, USA) was performed in a subgroup of patients. The study protocol was approved by the local Ethics Committee.

### 2.2. Pulmonary Vein Isolation

All patients underwent preprocedural transthoracic echocardiography to asses left ventricular ejection fraction and left atrial dimension. To exclude the presence of thrombi in the left atrium or in the left atrial appendage a transesophageal echocardiography was performed the day before the procedure. Moreover, a preprocedure magnetic resonance imaging or computed tomography with segmentation of the left atrium was performed to assess left atrial anatomy in detail. Procedures were performed either with continued oral anticoagulation using warfarin and therapeutic INR (2.0 to 3.0) or using low-molecular weight heparin bridging. All PVI procedures were performed by experienced operators beyond the learning curve. Briefly, all procedures were carried out in conscious sedation using propofol infusion. A deflectable decapolar catheter was inserted through right femoral vein and positioned into the coronary sinus to guide the transseptal puncture and to pace the left atrium during treatment of the left PVs and was subsequently moved to the superior vena cava where it was used to stimulate the right phrenic nerve during treatment of the right PVs. A single transseptal puncture was performed using a needle system (BRK, St. Jude Medical, St. Paul, MN, USA) and a standard transseptal sheath (SL0 8F or 8.5F, St. Jude Medical, St. Paul, MN, USA), subsequently exchanged with a steerable 15F sheath (FlexCath*™*, 15F, Medtronic, Inc., Minneapolis, MN, USA). Before transseptal puncture, heparin was administered intravenously as bolus (10000 U) followed by a continuous infusion (1000 U/hr) reaching ACT level >350 sec. The FlexCath was continuously irrigated with heparinized saline (2 mL/hr). An esophageal temperature probe was used in all patients (Esotherm Plus, FIAB) to monitor intraesophageal temperature increase. The probe was adjusted during the procedure to stay as close as possible to the ablation catheter. Cryotherapy was interrupted if the endoluminal esophageal temperature dropped below 18°C. Two cryotherapy applications were delivered to each PV, 240–300 seconds each, aiming for a minimum temperature of less than −40°C. After treatment of all PVs, entrance block was confirmed with high-output pacing (12 V, 2.9 ms) using the Lasso*™* (Biosense Webster, Diamond Bar, CA, USA), Afocus (St. Jude Medical, Minneapolis, MN, USA), or Achieve*™* mapping catheter (Medtronic, Inc., Minneapolis, MN, USA). “Far field” capture and sensing were ruled out using differential pacing maneuvers. Any residual conduction into the PVs was treated by further cryotherapy applications. Successful PVI was confirmed when all PV potentials were abolished or were dissociated at least 20 minutes after the last cryotherapy application to that vein.

### 2.3. Lesion Area Comparison

**Figure 1:**
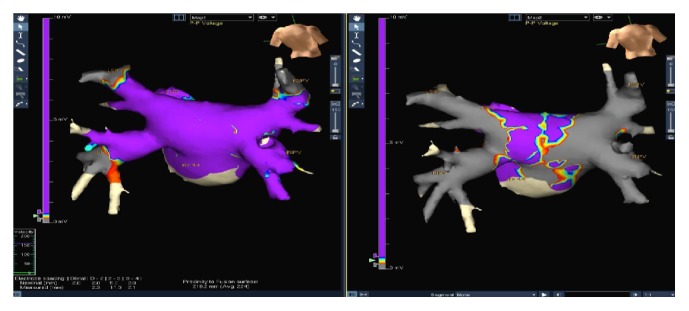
High-density voltage map of the left atrium using electroanatomic mapping, NavX Velocity 3.0, before and after the procedure.

In each patient who underwent PVI using the electroanatomic mapping system NavX Velocity 3.0, a high-density voltage map of the left atrium was performed, before and after the procedure, using the mapping catheter Afocus. After cryotherapy, the border between the scar area and healthy atrial tissue was defined using a 0.1–0.5 mV as offset (0.1 mV was defined as scar or absolutely silent tissue). The border between scar and normal tissue was defined including both ipsilateral PVs. Using an implemented tool in the NavX Velocity 3.0, the lesion area (cm^2^) was automatically calculated by the system (Figure 1).

### 2.4. Follow-Up

Patients were followed up in the outpatient clinic 3 months after the procedure and every 3 months during the first year after ablation and every 6 months thereafter. At each visit, a standard 12-lead ECG was obtained in all patients. All patients were followed up with Holter-ECG monitoring at 6 and 12 months and annually after the PVI procedure. After 90 days of blanking period, any documented episode of AF or atrial arrhythmias lasting >30 seconds was considered a recurrence. All antiarrhythmic agents were withdrawn at 3 months after ablation. Clinical events occurring during the follow-up and documentation of the events were carefully checked. Clinical success was defined as complete freedom from symptomatic arrhythmia and the absence of any atrial arrhythmia during Holter monitoring.

### 2.5. Statistical Analysis

This was an observational, retrospective, single-center study. Continuous variables are reported as mean ± standard deviation. Comparison of continuous variables was performed using independent sample Student's* t*-test and categorical data with Fisher's exact test. Arrhythmia-free survival curves were generated by the Kaplan-Meier method and compared with the Log Rank test. Statistical significance was considered with a *p* value of <0.05. SPSS 20.0 statistical software (SPSS Italia, Inc., Florence, Italy) was used for statistical analysis.

## 3. Results

Baseline clinical characteristics of patients are reported in Table 1. There were no significant differences between the 2 study groups regarding age, gender, cardiovascular risk factors, left ventricular ejection fraction, left atrial dimension, and medical therapy. No significant differences were found between the two study groups regarding CHA2DS2-VASc and HAS-BLED scores.

**Figure 2:**
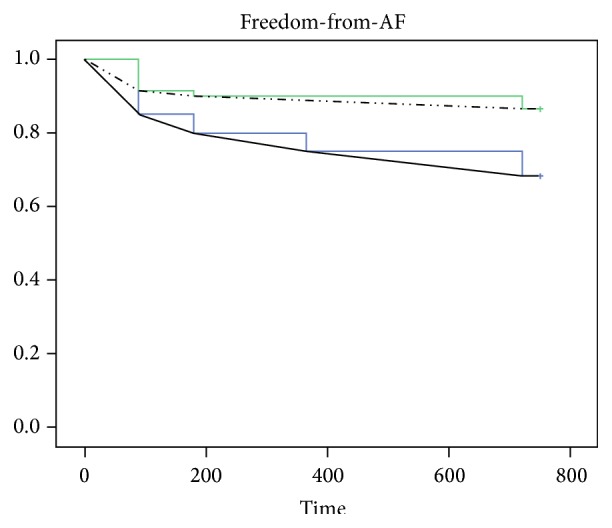
The Kaplan-Meier survival analysis shows a significant difference in freedom-from-AF recurrence between patients undergoing atrial fibrillation ablation using the first-generation Cryoballoon (CB1) and the second-generation Cryoballoon (CB2) catheter (Log Rank *p* = 0.017).

No patients had evidence of left atrial thrombosis during transesophageal echocardiography. Acute success rate and procedural-related complications are reported in Table 3. Procedures performed with the first generation CB showed longer fluoroscopy time (36.3 ± 16.8 versus 14.2 ± 13.5 min, resp.; *p* < 0.001) and longer procedure times as well (153.1 ± 32 versus 102 ± 24.8 min, resp.; *p* = 0.019) compared to the second-generation ADV catheter (Table 2). Interestingly, no statistically significant differences were found in the lesion area of left and right PVc between the two groups (resp., *p* = 0.61 and 0.57, Table 4). The overall success rate after single PVI procedure including both first- and second-generation CB was 77.5%. The long-term freedom-from-AF as showed in the Kaplan-Meier survival analysis was significantly different between the two different CB (68.3% with the first-generation CB versus 86.7% with the second-generation ADV catheter, resp.; Log Rank *p* = 0.017, Figure 2).

## 4. Discussion

This retrospective analysis provides data on long-term efficacy of CB ablation performed in a single high-volume center. The main findings of this study are that the use of the second-generation ADV catheter significantly improved the long-term procedural success after single PVI procedure and reduced procedure duration and fluoroscopy exposure time.

Our results in terms of procedural success using the first generation CB are in line with those coming from the North American Arctic Front STOP AF Pivotal Trial (68.3% versus 69.9% resp.) [13]. Several reports have shown that CB ablation with the new ADV catheter is associated with higher success rate of PVI and better outcome. In a first report of Fürnkranz et al. comparing the first-generation CB with the ADV, single-shot PVI rate increased from 51% to 84% (*p* < 0.001) [12]. Procedure duration and fluoroscopy exposure time were also significantly decreased using the novel CB catheter. In a retrospective analysis, Aryana et al. confirmed that ADV catheter significantly reduced procedure time and fluoroscopy time. Freedom from AF at 6, 9, and 12 months was 89, 86, and 82%, respectively, during a mean follow-up of 16 ± 8 months [14]. Giovanni and coworkers recently reported a significantly higher freedom from AF at 1-year follow-up with the second-generation ADV catheter with respect to the first-generation CB. Freedom from AF off antiarrhythmic drugs (AAD) therapy was achieved in 84% of patients treated with the ADV catheter, compared to 66% of success rate obtained with first-generation CB (*p* = 0.038). In their experience, procedural and fluoroscopy times were also significantly decreased by the use of ADV catheter [15]. Similar findings were reported by Fürnkranz et al. The authors found freedom from AF after a single procedure without AAD therapy after 1 year in 63.9% of patients treated with the first generation of CB versus 83.6% (*p* = 0.008) of patients with the ADV catheter [16]. Liu et al. during a mean follow-up of 12 ± 4 months found an overall 76.0% of CB success rate, respectively, 89.7% with ADV catheter versus 59.7% with the first-generation CB (*p* < 0.001) [17]. In addition to previous published paper, we performed for the first time a comparison between lesion areas created by the two different CB. Despite the redesign of the ADV catheter, in our experience the improved acute and long-term procedural success seems not to be related to an increased area of lesion. The main technical limitation of the first generation CB was the temperature gradient from the equator to the distal pole of the CB. More specifically, the first-generation CB had four injection ports positioned just distal to the equator, cooling the balloon surface with a temperature gradient with relatively higher temperatures at the distal pole. As a result, continuous lesions are created if the balloon is centered in the PV antrum. Differently, eccentric CB positions may lead to incomplete lesion formation of tissue, resulting in reconnection gap. Thus, repeated freezing with different CB positions were often necessary to achieve PVI prolonging both procedural and fluoroscopy time. The ADV catheter was redesigned doubling the injection ports and placing themselves more distally on the catheters shaft creating a larger and more uniform zone of freezing on the CB surface. Together, these modifications have been shown to improve procedural and early clinical efficacy during short-term follow-up. Notably, we report a very low incidence of procedure-related complications. It could be related to the size of CB used at our center; indeed we only use the 28 mm balloon due to safety reasons. Creation of proximal lesions at the antrum of PVs should prevent or at least reduce complications such as PV stenosis and phrenic nerve palsy.

## 5. Study Limitations

This study has some limitations: it is a single-center retrospective analysis in a highly selected population. In order to complete at least 2 years of follow-up, we excluded patients in which the follow-up was not fully available. Finally, the follow-up was performed for the majority of patients with 12-lead ECG and Holter-ECG monitoring; unfortunately, an event recorder was not available for all patients.

## 6. Conclusion

On the long-term follow-up, PVI using the ADV performs significantly better when compared to the first-generation CB. Procedure duration and fluoroscopy exposure time were significantly shortened with the ADV catheter. Based on electroanatomical mapping, lesion areas created by the two CB were not statistically different.

## Figures and Tables

**Table 1:** Baseline patient characteristics.

	CB, 1st (*n* = 60)	CB, 2nd (*n* = 60)	*p*
Male sex, *n* (%)	41 (68.3)	50 (83.3)	0.14
Mean age, years (mean ± SD)	59.1 ± 12.2	57.2 ± 10.9	0.37
Body mass index, Kg/m^2^ (mean ± SD)	26 ± 2	26 ± 3	0.59
Paroxysmal atrial fibrillation, *n* (%)	60 (100)	60 (100)	1
Left atrial diameter, mm (mean ± SD)	22.9 ± 5.1	22.5 ± 4.7	0.60
Left ventricular ejection fraction, (mean ± SD)	62.5 ± 6.1	60.9 ± 7.4	0.72
Hypertension, *n* (%)	25 (41.6)	23 (38.3)	0.63
Hypercholesterolemia, *n* (%)	12 (20)	14 (23.3)	0.61
Diabetes mellitus, *n* (%)	4 (6.6)	5 (8.3)	0.73
Hypertriglyceridemia, *n* (%)	5 (8.3)	6 (10)	0.71
Active smoking, *n* (%)	8 (13.3)	9 (15)	0.69
Coronary artery disease, *n* (%)	4 (6.6)	5 (8.3)	0.73
Dilated cardiomyopathy, *n* (%)	0	0	—
Valve disease, *n* (%)	4 (6.6)	3 (5)	0.40
Previous cardiac surgery, *n* (%)	3 (5)	2 (3.3)	0.46
Previous ischemic stroke, *n* (%)	—	1 (1.6)	0.53
Chronic renal failure, *n* (%)	4 (6.6)	3 (5)	0.40
Previous ablation procedures for AF, *n* (%)	0	0	—

**Table 2:** Fluoroscopy time and procedure time comparison between the first- and second-generation CryoBalloon catheter.

	CB, 1st	CB, 2nd	*p*
Procedure time, min (mean ± SD)	153.1 ± 32	102 ± 24.8	0.019
Fluoroscopy time, min (mean ± SD)	36.3 ± 16.8	14.2 ± 13.5	<0.001

**Table 3:** Acute success and procedure-related complications.

	CB, 1st (*n* = 60)	CB, 2nd (*n* = 60)	*p*
PVI achieved, (%)	95	98	ns
Catheter failure, *n* (%)	3^*∗*^ (5)	1^#^ (1.6)	ns
Need of touch-up, *n* (%)	3 (5)	1 (1.6)	ns
Acute PNP, *n* (%)	2 (3.3)	1 (1.6)	ns
Chronic PNP, *n* (%)	0	0	—
Cerebral embolization, *n* (%)	0	0	—
Pericardial effusion, *n* (%)	1 (1.6)	0	ns
Cardiac tamponade, *n* (%)	0	0	—
PV stenosis, *n* (%)	0	0	—
Atrioesophageal fistula, *n* (%)	0	0	—
Vascular injury, *n* (%)	3 (5)	2 (3.3)	ns

PVI: pulmonary vein isolation; PNP: phrenic nerve palsy; #: FlexCath failure; *∗*: 2/3 FlexCath failure, 1/3 Cryoballoon failure.

**Table 4:** Comparison of lesion area between the first- and second-generation CryoBalloon catheter. Data obtained from electroanatomic mapping performed after cryoablation using the NavX system (St. Jude Medical, St. Paul, MN, USA).

Lesion area	CB, 1st	CB, 2nd	*p*
LPVs, cm^2^ (mean ± SD)	68.2 ± 44	75.3 ± 26	0.61
RPVs, cm^2^ (mean ± SD)	73.1 ± 33	79.4 ± 22	0.57

LPVs: left pulmonary veins; RPVs: right pulmonary veins.

## Abbreviations

PVI: Pulmonary vein isolation
PAF: Paroxysmal atrial fibrillation
CB: Cryoballoon
ADV: Arctic front advance
PV: Pulmonary vein
AAD: Antiarrhythmic drug.
